# Royal Medical and Chirurgical Society

**Published:** 1897-02-13

**Authors:** 


### ROYAL MEDICAL AND CHIRURGICAL SOCIETY.

Dr. John Abercrombie read a paper on "Some Affections of the Nervous System met with in Association with an Attack of Enteric Fever." He drew attention to the occasional occurrence of imbecility after enteric fever, and endeavoured to show that a lack of sufficient nourishment during the attack was probably a main factor in its causation. Reference was also made to the development of rigors during or after the attack, mostly associated with intestinal irritation, but in one case with venous thrombosis. Two cases were related in which convulsions occurred in the course of enteric fever, both patients being adults; in the first case they were due to intestinal irritation; in the second to the passage of a portion of thrombus to the heart.

In discussing these cases Dr. Herringham drew attention to the condition of instability of the nervous system which came on in the later stages of typhoid fever, as shown by the oscillations and variations in the temperature. A rigor had a certain analogy with an epileptic fit, and he thought that both rigor and convulsion might occur without any definite physical cause. In some cases the rigor might coincide with the pyrexial point of a temperature curve, but, on the other hand, the high temperature might occur without any rigor coming on. He did not, however, think that this condition of nervous system was to be put down to defective feeding, for such conditions were met with where the patients had been well treated in hospitals. The best food that such a patient could digest would not always give the nervous system proper nourishment during the prolonged course of such a disease as typhoid fever.

Mr. Norman Moore thought that although it was true enough that rigors might be set up by intestinal irritation, it should never be forgotten that they might mean serious mischief; and he related two cases in which rigors, just such as had been described, were followed by the discharge of pus in the urine, showing that there had been deep-seated abscess. It also should be remembered that a rigor occurring during recovery from typhoid fever might mean that an old tuberculous deposit had started into fresh activity. Rigors, therefore, could never be looked on lightly.

Sir Dyce Duckworth pointed out the great variety of infections, primary and terminal, which were concerned in the production of the sum total of the symptoms of typhoid fever. In regard to the mental enfeeblement which was apt to follow this disease, he questioned whether it was anything special to enteric fever, or differed from what might arise from defective nutrition from any febrile illness in which the feeding was interfered with for so long a time. Both mania and melancholia might be met with, and might be permanent, but it was to be noted that under such circumstances it was generally due to inherited tendencies, and that the typhoid fever was merely the means by which these tendencies were brought into activity.

Dr. Horton-Smith read a paper on "The Presence of Typhoid Bacilli in the Urine." Since the year 1886 various papers had appeared stating that typhoid bacilli occurred with great frequency in the urine of patients suffering from typhoid fever. The writers of these papers seemed to have found them with great ease, and sometimes as early as the third day. Unfortunately, however, these results, which, if true, would be of extreme importance, could not be accepted as of much value at the present day, for the simple reason that the observers quoted above had not had the means which we now possess for distinguishing the typhoid bacillus from others resembling it, and especially from the Bacillus Coli, a bacillus which was found by no means unfrequently in urine. Hence, all statements made hitherto as to the presence of typhoid bacilli in urine fell to the ground.

